# Comparison of Retention between Conventional and Nanofilled Resin Sealants in a Paediatric Population: A Randomized Clinical Trial

**DOI:** 10.3390/jcm11123276

**Published:** 2022-06-08

**Authors:** Vinayak Kamath, Mamata Hebbal, Anil Ankola, Roopali Sankeshwari, Sagar Jalihal, Abhra Choudhury, Mai Soliman, Elzahraa Eldwakhly

**Affiliations:** 1 Department of Public Health Dentistry, Goa Dental College and Hospital, Bambolim 403202, India; b_vk2002@yahoo.com; 2 Department of Preventive Dental Sciences, College of Dentistry, Princess Nourah Bint Abdulrahman University, P.O. Box 84428, Riyadh 11671, Saudi Arabia; mihebbal@pnu.edu.sa; 3 Department of Public Health Dentistry, KLE VK Institute of Dental Sciences, Belagavi 590010, India; dranilankola55@gmail.com (A.A.); docrups@gmail.com (R.S.); drsagarjalihal@gmail.com (S.J.); abhra.rkmv@gmail.com (A.C.); 4 Department of Clinical Dental Sciences, College of Dentistry, Princess Nourah Bint Abdulrahman University, P.O. Box 84428, Riyadh 11671, Saudi Arabia; msmustafa@pnu.edu.sa

**Keywords:** prevention, nanofilled resin, primary school children, sealant retention

## Abstract

Background: This study compared conventional-resin-sealant versus nanofilled-sealant retention at different intervals. Methods: A double-blinded split-mouth randomized control trial was performed on sixty-two children aged from six to nine years. Participants with one pair of contralateral permanent first molars with deep fissures or noncavitated carious lesions were randomly selected for sealant application. Conventional resin sealant was applied on one molar and nanofilled sealant on the contralateral molar. Evaluations were performed at one, three, six, twelve and eighteen months to check for retention. The chi-squared test, McNemar test, Wilcoxon signed-rank test and Friedman test were used for statistical analysis. Results: Conventional resin sealant showed complete retention in 91.4%, 86.2%, 74.1%, 62.1% and 55.2% of the teeth, and nanofilled sealant showed complete retention in 89.7%, 81%, 77.6%, 69% and 67.2% of the teeth, at the end of 1 month, 3 months, 6 months, 12 months and 18 months evaluation, respectively. Each sealant exhibited a statistically significant change (*p* < 0.05) in the retention rate during the evaluation period. However, when both the sealants were compared with each other, there was no statistically significant difference in any phase. At the end of 18 months, caries development was observed in 13.8% of the teeth sealed with conventional sealant, and in 10.3% of the teeth sealed with nanofilled sealant. Conclusion: At eighteen months, the nanofilled resin sealant exhibited complete retention in 12% more teeth than the conventional sealant. However, the difference was not statistically significant. The nanofilled resin sealant yielded an acceptable performance in sealing the occlusal pits and fissures of mandibular permanent first molars, compared to conventional pit-and-fissure sealants.

## 1. Introduction

Dental caries is undoubtedly one of the most frequently encountered problems related to oral health, and especially among children. The action of bacteria on food particles results in acid production, which results in the gradual breakdown of enamel, which causes dental caries [1]. According to the Global Burden of Disease 2010 study, caries in permanent teeth is the most prevalent condition, affecting 35% of the global population, and caries in primary teeth ranks tenth amongst the most prevalent conditions, affecting 9% of the global population [2]. The most common occlusal caries is present in the pits and deep fissure-like areas of young permanent posterior teeth. Pits and deep fissure-like areas are basically occlusal anomalies that are seen at the development groove junctions and in between the cusps, respectively [3]. Food particles are trapped in pits and fissure easily, acting as initiators of dental caries [4]. Dental caries, due to its high prevalence, leads to huge economic costs, both direct (dental treatment cost) and indirect (loss of productivity) [5]. Thus, it becomes a major public health problem, as the expenses have to be covered from one’s pocket or by the government, which poses an extra burden on the financial strains of countries [6].

The first molar is usually the first tooth to erupt in young permanent dentition, and thus it is the most susceptible to decay within the first 2–4 years of its eruption [7]. Children find it difficult to practice proper oral hygiene measures, and especially posteriorly, as it is relatively difficult to access [8]. Previously, the strategies to prevent pit-and-fissure caries included blocking the pit and fissures by using zinc phosphate cement, mechanical fissure eradication, prophylactic odontotomy and chemical treatment with silver nitrate. In 1955, Buonocore introduced acid etch bonding to enamel, which, together with the Bis-GMA (bisphenol A-glycidyl methacrylate) material developed by Bowen in 1962, led to the birth of pit-and-fissure sealants [9]. Ever since, pit-and-fissure sealants have become the most commonly accepted treatment modality to prevent occlusal caries [10]. The cariostatic properties of pit-and-fissure sealants are mainly attributed to the physical obstruction of pits and fissures, as they remain tightly bonded to the surface of enamel. This prevents the bacterial colonization of pits and fissures, as well as the inhibition of the availability of fermentable carbohydrates to any bacteria remaining in the pits and fissures [11]. When pit-and-fissure sealants are applied at a young age, they protect the permanent teeth, and especially the occlusal surface, for a longer period of time. A study by Okunseri CE et al. reveals that children who received pit-and-fissure-sealant placement on their young permanent first molars had less of a requirement for subsequent restorative treatment [12]. Several materials have been suggested for use since 1967, but the most common pit-and-fissure-sealant materials used today are resin-based sealants and glass ionomer cements. Several studies have shown that resin-based sealants are very effective in the permanent teeth of children and adolescents, making them the material of choice for sealing pits and fissures [13].

The success of the pit-and-fissure sealant is mainly dependent on its retention on the occlusal surface [14]. Other important factors are its biocompatibility with the oral tissues, low solubility in the oral cavity, good bond strength, anticariogenic nature, and adequate marginal adaptation. The retention of the sealant is primarily influenced by its flow characteristics. A sealant with a good flow property can penetrate the narrow pits and fissures and has better retention [15]. However, rarely are sealants retained completely over the tooth’s lifetime, and reapplication is required. Filler material, usually consisting of quartz and silica particles, is added to the resin to increase the bond strength and the resistance to abrasion and wear. The applicability of flowable composites has increased in recent years, mainly because of their beneficial properties, such as low viscosity, low modulus of elasticity and ease of handling, which helps in the successful placement of the material. Moreover, higher filler particle amounts provide lesser porosity and better wear resistance compared to those of conventional resin pit-and-fissure sealants [16]. However, clinicians believe that increasing the filler content leads to an increase in the viscosity and lower retention. With the development of nanofillers, it has become possible to increase the filler content in composite resins without adversely affecting the flow properties [17]. Due to the increased filler content, mechanical properties, such as the strength and wear resistance, could also be improved.

In the present study, a conventional resin sealant, Delton FS+ (Dentsply Professional, York, PA, USA), was compared to a newer nano-filled flowable composite, Filtek Z350 (3M ESPE, St. Paul, MN, USA). Delton is a light-cured filled resin-based sealant that is commonly used for fissure sealing. Filtek Z350 is a flowable nanocomposite that is indicated as a pit-and-fissure sealant. This study attempted to evaluate and compare the effectiveness and retention ability of conventional and nanofilled resin sealants.

## 2. Materials and Methods

A randomized control clinical trial using a split-mouth technique was performed among six-to-nine-year-old primary school children in Belagavi City, India. The study commenced on January 2011 (when the participants were screened and selected on the basis of the selection criteria) and continued till October 2012. It was carried out in accordance with the Code of Ethics of the World Medical Association (Declaration of Helsinki). Ethical approval was obtained prior to conducting the study from the Research and Ethical committee of the Institute of Dental Sciences, and the Deputy Director of Public Instruction (DDPI), Belgaum City. Written informed consent was obtained from parents of all children who participated in the study. Principals/head masters of the schools also consented to the study.

On the basis of the expected difference in the effectiveness between the two sealants, a sample size of 56 children was calculated. Assuming 10% dropout, the final sample size was increased to 62 primary school children, with a total of 124 teeth (62 pair sites).

Inclusion criteria for children included: (a) decay–filled-teeth (dft) score of 3–6; (b) the presence of at least one pair of contralateral permanent mandibular first molars with deep fissures or noncavitated carious lesions; (c) the entire occlusal surface was exposed, without the presence of operculum covering the tooth. Exclusion criteria included: (a) teeth with discolouration, restorations, cavitations or developmental defects; and (b) children who were physically and mentally challenged, or with gross orofacial defects or debilitating diseases.

The list of all primary schools located in Belagavi city was taken from the Deputy Director of Public Instruction (DDPI), and two schools were randomly selected from it using the lottery method to comprise the two groups of children, aged between six and nine years old. A proforma was self-designed and it included basic information, such as sociodemographic details, dietary habits and the practices related to the maintenance of oral hygiene.

The principal investigator (VK) performed the clinical examination of every participant and the application of pit-and-fissure sealant, and the coinvestigator (MH) conducted the evaluation of the sealant during the follow-up period. Prior to conducting the study, the calibration of the investigator to screen the participants and perform the sealant application was performed. The criteria for caries diagnosis, the indication for sealing and the criteria for assessment of sealant were discussed. A clerk was trained to record the findings and assist the investigator in whatever way possible.

The study was conducted in 3 phases:

(1) Screening phase

During the screening phase, the examination was conducted on the school premises. The schoolteachers and children were informed about the purpose of the study and the procedures that would be undertaken. The self-designed proforma was filled by interview method. For each visit, 30 sets of autoclaved mouth mirrors, explorers and tweezers were made available. A Type III examination was performed by making the participants sit comfortably on a chair and using a mouth mirror and explorer under natural lighting conditions. The recording clerk noted down the findings.

In total, 218 students from six to nine years old underwent the process of screening in the initial stage. Among them, 93 students met all the eligibility criteria. Random selection was conducted among them using the lottery method to finally select 62 students with 62 site pairs.

(2) Sealant application phase

After obtaining approval from the school authorities and informed consent from the parents, five students were brought to the dental hospital on a daily basis. The procedure was performed in a dental clinic using the two-hand technique. This is a double-blinded study, wherein the participants and evaluator during follow up were not aware as to which teeth in the split mouth received conventional sealant and which received nanofilled sealant. The investigator who applied the sealant could not be blinded, as the sealant materials had different packaging and the application techniques were different. The tooth on which conventional sealant was to be applied was randomly selected by the lottery method. The contralateral tooth was selected for nanofilled resin sealant. For the next participant, the sealant-application order of the tooth was reversed, and it was carried out in the same manner alternatively.

Prior to the application of the sealant material, the pit and fissures of the selected tooth were examined for any debris, and, if any, it was removed using sickle explorer, forcefully rinsed with water and cleaned with wet and dry cotton pellets. For all sealant applications, isolation of the teeth was performed using cotton rolls and suction. Both the materials were applied following the manufacturer’s instructions.

The material used as conventional resin sealant was Delton FS+ (Dentsply Professional, York, PA, USA). The material used for nanofilled sealant was Filtek Z350 flowable nanocomposite (3M ESPE, St. Paul, MN, USA).

Procedure for conventional resin sealant:

The material used as conventional resin sealant was Delton FS+ (Dentsply Professional, York, PA, USA). The tooth was etched using ‘Delton EZ etch’ etchant gel, as per the manufacturer’s instructions, for 30–60 s, followed by thorough rinsing for at least 30 s. Etching was confirmed by a dull frosty white appearance of the enamel. If salivary contamination occurred, the surface was again cleaned, dried and re-etched. The resin material was introduced into the etched pit and fissures of the isolated tooth using a disposable brush-tip applicator. The sealant material was then cured using visible light, as per the manufacturer’s instructions. The sealed area was checked with an explorer for complete coverage and retention. The occlusion was checked with articulating paper and was adjusted, if necessary, with a finishing bur.

Procedure for nanofilled sealant:

The material used for the nanofilled sealant was Filtek Z350 flowable nanocomposite (3M ESPE, St. Paul, MN, USA). A single-component self-etching light-cure adhesive was used as the etching-and-bonding agent, as per the manufacturer’s instructions. The sealant material was introduced into the etched and bonded pits and fissures of the isolated tooth using a syringe applicator. The sealant material was then cured for 40 s using visible light, as per the manufacturer’s instructions. The sealed area was checked with an explorer for complete coverage and retention. The occlusion was checked with articulating paper and adjusted, if necessary, with a finishing bur.

(3) Evaluation phase

Evaluation was conducted at 1 month, 3 months, 6 months, 12 months and 18 months after placement of the sealant for retention and caries incidence. Sealants were assessed for coverage and caries incidence according to the Color, Coverage, Caries (CCC) Sealant Evaluation System, described by Deery C et al. [18]. The examination was performed on the school premises under natural lighting conditions.

Statistical Analysis:

The data collected were entered into a Microsoft Excel spreadsheet and analyzed using IBM SPSS Statistics, Version 22 (IBM Corp., Armonk, NY, USA). For inferential statistics, the chi-squared test, McNemar test, Wilcoxon signed-rank test and Friedman test were used. *p* value < 0.05 was considered statistically significance.

## 3. Results

Out of the 62 participants that were included in the study, 4 participants were lost to follow up. The 58 children that were evaluated over 18 months consisted of 34 (58.6%) boys and 24 (41.4%) girls. The distribution of the study participants by age and gender is presented in Table 1.

The present study followed a split-mouth design. The teeth distributions of the conventional resin sealant and the nanofilled resin sealant are given in Table 2. In each of the 58 study participants, two teeth (i.e., mandibular first permanent molar on both third and fourth quadrants) were included. Conventional resin sealant was used to seal 27 (46.6%) left first mandibular molars (#36), and 31 (53.4%) right first mandibular molars (#46). Nanofilled sealant was used to restore each of the opposing contralateral first mandibular molars.

The distribution of the study participants, according to the retention of the conventional sealant, is given in Table 3. When the retention of the conventional sealant was evaluated after one month of application, as per the evaluation criteria, 53 (91.4%) sealants were completely retained in all tested fissures. At three months, 50 (86.2%) conventional sealants were completely retained, and 43 (74.1%) sealants were retained at six months evaluation. At 12 months and 18 months evaluation, 36 teeth (62.1%) and 32 teeth (55.2%) had complete retention of the sealant, respectively. A statistically significant change in the retention rate of the conventional sealant was observed at one, three, six, twelve and eighteen months evaluation.

The distribution of the study participants according to the retention of the nanofilled resin sealant is presented in Table 4. When the retention of the nanofilled resin sealant was evaluated after one month, 52 (89.7%) sealants were completely retained in all tested fissures. At 3 months, 6 months, 12 months and 18 months, the nanofilled sealants in 47 teeth (81%), 45 teeth (77.6%), 40 teeth (69%) and 39 teeth (67.2%), respectively, were completely retained. A statistically significant change in the retention rate was observed at one, three, six, twelve and eighteen months evaluation.

There was no significant difference in the retention levels between conventional and nanofilled resin sealants at the end of each evaluation period (Table 5).

During 1 and 3 months of evaluation, no caries was detected in any of the sealed teeth in both the conventional-sealant group and the nanofilled-resin-sealant group. At the end of 6 months and 12 months, 55 (94.8%) and 52 (89.7%) of the teeth sealed with conventional resin sealant were found to be caries-free, respectively, and caries had developed in 4 (5.2%) teeth and 6 (10.3%) teeth, respectively. At the same time, 55 (94.8%) teeth and 53 (91.4%) teeth sealed with nanofilled resin sealant were also found to be caries-free at 6 months and 12 months evaluation, respectively, and caries had developed in 4 (5.2%) teeth and 5 (8.6%) teeth, respectively. At the end of 18 months, 50 (86.2%) teeth sealed with conventional resin sealant were found to be caries-free, and caries had developed in 8 (13.8%) teeth. At the same time, 52 (89.7%) teeth sealed with nanofilled resin sealant were also found to be caries-free, and caries had developed in 6 (10.3%) teeth. There was no statistically significant difference (*p* > 0.05) in the caries occurrence in the teeth sealed with conventional and nanofilled resin sealants compared at the end of 6 months, 12 months and 18 months (Table 6).

## 4. Discussion

Pit-and-fissure caries has been a cause for concern for a long time. The first tooth surface that is generally affected by caries in a young child is the occlusal surface of the permanent first molar. The complex morphology of occlusal surfaces makes the mechanical removal of dental plaque difficult and allows for plaque accumulation. In this context, pit-and-fissure sealants have been considered an important adjunct to oral healthcare strategies and fluoride therapy in preventing occlusal carious lesions [13].

This study used a split-mouth design, where a tooth on one side of the mouth was sealed with conventional resin sealant while the contralateral tooth was sealed with nanofilled sealant. The split-mouth design has been used in various studies conducted by Fernández-Barrera MÁ et al. [19] and Naaman R et al. [9] for comparing pit-and-fissure sealants. This design helps to control many potentially confounding factors, and particularly those due to the physiological characteristics of the participants, dietary and oral hygiene habits and preventive regimes.

The present study shows that the loss of retention of the conventional sealant at the end of one month was 8.6%, which increased to 13.8% in three months, and to 25.9% by six months. This was much higher when compared to studies that used similar materials. A study conducted by Yan WJ et al. [20] reports a retention loss of 8.69% at six months. Similarly, P Kumaran [21] and Jafarzadeh M et al. [15] report lower retention loss.

At six months, the loss of retention of the nanofilled resin sealant was 22.5%, which was comparatively less than that of the conventional sealant, but there was no statistically significant difference. This retention loss was much higher when compared to the studies using flowable composites by Amin HE et al. [22], which report a (10%) loss of sealant retention. However, due to the major lack of adequate published literature, appropriate comparisons cannot be drawn.

The complete retention at 12 months was 62.1% for Delton and 69% for Filtek. These findings are lower when compared to the study conducted by Jafarzadeh M et al. [15], where the complete retention at the end of 12 months was 89.7% for the flowable composite and 84.6% for the conventional sealant. According to a study by Yazici AR et al. [23], the retention at the end of 12 months was 89.3% using a nanofilled resin sealant. Similarly, in a study conducted by Perveen Z et al. [24], the retention was higher in nanofilled sealant (84.7%) when compared to filled resin sealant (74.3%). A study conducted by Corona SA et al. [25] also reports the complete retention of conventional sealant in 95% of teeth, and all teeth sealed with flowable composite showed complete retention over one year of follow up. A 48-month evaluation study conducted by Kamaran E et al. [26] reveals a retention rate of 89.3% for a nanofilled resin sealant. A major reason for the high retention rates observed in the studies mentioned above could be due to the usage of rubber dams to achieve isolation and a more rigorous surface preparation.

In our study, at 18 months evaluation, 55.2% of the conventional sealants showed complete retention, and 67.2% of the nanofilled sealants showed complete retention. When compared to the results of 24 months evaluation by Yakut N et al. [27], the rate of retention of the conventional sealant achieved in this study was a low recording (90%) of retention. In comparison, the retention of the nanofilled sealant was better than the conventional sealant, but still low. Sealant retention loss could be due to various reasons, such as: poor placement technique; inadequate moisture control; not sealing all pits/fissures; inadequate etching, rinsing and drying; insufficient curing time; material wear; and/or a combination of these factors. Other variables that influence the sealant retention are the position of the tooth in the mouth, the skill of the operator and the age of the patient [28]. Moreover, the clinical evidence suggests that the loss of sealant retention occurs due to a number of reasons. Initially, it could be due to a faulty technique, such as moisture contamination. Secondly, the loss could be due to material wear under the forces of occlusion, which could be further compounded by the improper selection of the tooth. In the present study, it can be assumed that tooth selection or technique failure at the time of sealant placement could be responsible for the majority of the retention failure that occurred within the first six months of the placement.

At the start of the study, the sealant application procedure was demonstrated before the study participants on a dummy model in order to acquaint them with the various steps involved. This helped to allay the fear and improved the co-operation of the participants. To avoid operator variability, a single investigator administered all the sealant applications. To avoid operator fatigue, a limited number of sealant applications was performed daily. By the end of 18 months, 4 participants out of 62 dropped out as the family had to move out of the area. Since, at the start of the study, the sample size was determined by assuming 10% as dropouts, the loss of four participants during follow-up did not pose a problem for assessing the study results.

The limitation of this study is the short evaluation period of 18 months that was used to assess the caries-preventive effect of the sealants. Hence, studies with longer-term follow-up periods are recommended to assess the retention and caries development. The conventional and nanofilled resin sealants were both applied by using two different techniques. Hence, the variations observed in the outcome could be due to the differences in the techniques used. Although the utmost care was taken to achieve adequate isolation and to prevent moisture contamination, the usage of rubber dams could have contributed to the improved retention rate in the present study. It cannot be estimated, from the present study, whether the reapplication of sealants increases the caries-preventive effect of the sealant, since only a single application of the sealant was carried out. During screening, noncavitated carious teeth were also included with the sound category, as per the guidelines for the sealant, and the pit-and-fissure sealant was applied. A separate record of the total number of noncavitated carious teeth included at baseline, and their allocation to each group, was not maintained. An unequal distribution of them might lead to variation in the occurrence of caries between the groups. Various nanofilled flowable composite materials are available on the markets, which are recommended for pit-and-fissure sealant application. However, they have wide disparities in the formulation, characteristics and physical properties. Hence, long-term in vivo investigations must be performed to assess the feasibility and real benefits of using materials such as sealants.

## 5. Conclusions

At the end of 18 months, the nanofilled resin sealant had a higher percentage of teeth with the complete retention of the sealant at 67.2%, when compared to the conventional sealant at 55.2%, although the difference was not statistically significant. It may be concluded that the nanofilled resin sealant investigated in the present study yielded an acceptable performance in sealing the occlusal pits and fissures of the mandibular permanent first molar, when followed up over a period of 18 months, which is comparable to that of conventional pit-and-fissure sealants.

## Figures and Tables

**Table 1:** Distribution of study participants according to age and gender of the study population.

Age	Gender		Total
	Boys	Girls	
6	7 (63.6%)	4 (36.4%)	11 (19.0%)
7	6 (60.0%)	4 (40.0%)	10 (17.2%)
8	8 (50.0%)	8 (50.0%)	16 (27.6%)
9	13 (61.9%)	8 (38.1%)	21 (36.2%)
Total	34 (58.6%)	24 (41.4%)	58 (100.0%)
	Chi-squared value—0.705, df—3, *p* = 0.87 (NS)		

**Table 2:** Teeth-wise distribution of the conventional and nanofilled resin sealants.

Tooth Sealed	Sealant	
	Conventional Resin Sealant	Nanofilled Resin Sealant
Left first permanent molar (36)	27 (46.6%)	31 (53.4%)
Right first permanent molar (46)	31 (53.4%)	27 (46.6%)
Total	58 (100%)	58 (100%)

**Table 3:** Distribution of study participants according to retention of conventional sealant at one, three, six, twelve and eighteen months evaluation.

Coverage Code	Conventional Resin Sealant				
	1 Month	3 Months	6 Months	12 Months	18 Months
A	53 (91.4%)	50(86.2%)	43 (74.1%)	36 (62.1%)	32 (55.2%)
B	0	3 (5.2%)	9 (15.5%)	11 (19.0%)	12 (20.7%)
C	2 (3.4%)	0	1 (1.7%)	6 (10.3%)	8 (13.8%)
D	3 (5.2%)	5 (8.6%)	5 (8.6%)	5 (8.6%)	6 (10.3%)
Total	58 (100.0%)	58 (100.0%)	58 (100.0%)	58 (100.0%)	58 (100.0%)
	Chi-squared value *—69.254, *p*-value < 0.001				

* Friedman test. Code to indicate the area of the pit-and-fissure pattern covered: A—sealant present on all of fissure system; B—sealant present on >50% of fissure pattern but some missing; C—sealant present on <50% of fissure pattern; D—no sealant present.

**Table 4:** Distribution of study participants according to retention of nanofilled resin sealant at one, three, six, twelve and eighteen months evaluation.

Coverage Code	Nanofilled Resin Sealant.				
	1 Month	3 Months	6 Months	12 Months	18 Months
A	52 (89.7%)	47 (81.0%)	45 (77.6%)	40 (69.0%)	39 (67.2%)
B	2 (3.4%)	5 (8.6%)	6 (10.3%)	10 (17.2%)	7 (12.1%)
C	1 (1.7%)	1 (1.7%)	2 (3.4%)	2 (3.4%)	5 (8.6%)
D	3 (5.2%)	5 (8.6%)	5 (8.6%)	6 (10.3%)	7 (12.1%)
Total	58 (100.0%)	58 (100.0%)	58 (100.0%)	58 (100.0%)	58 (100.0%)
	Chi square value *—44.72, *p*-value < 0.001				

* Friedman test. Code to indicate the area of the pit-and-fissure pattern covered: A—sealant present on all of fissure system; B—sealant present on >50% of fissure pattern but some missing; C—sealant present on <50% of fissure pattern; D—no sealant present.

**Table 5:** Comparison of retention between conventional (Delton) and nanofilled (Filtek) resin sealant at one, three, six, twelve and eighteen months evaluation.

Coverage Code	1 Month		3 Months		6 Months		12 Months		18 Months	
	Delton	Filtek	Delton	Filtek	Delton	Filtek	Delton	Filtek	Delton	Filtek
A	53 (91.4%)	52 (89.7%)	50 (86.2%)	47 (81.0%)	43 (74.1%)	45 (77.6%)	36 (62.1%)	40 (69.0%)	32 (55.2%)	39 (67.2%)
B	0	2 (3.4%)	3 (5.2%)	5 (8.6%)	9 (15.5%)	6 (10.3%)	11 (19.0%)	10 (17.2%)	12 (20.7%)	7 (12.1%)
C	2 (3.4%)	1 (1.7%)	0	1 (1.7%)	1 (1.7%)	2 (3.4%)	6 (10.3%)	2 (3.4%)	8 (13.8%)	5 (8.6%)
D	3 (5.2%)	3 (5.2%)	5 (8.6%)	5 (8.6%)	5 (8.6%)	5 (8.6%)	5 (8.6%)	6 (10.3%)	6 (10.3%)	7 (12.1%)
*p* value *	1.000, NS		0.464, NS		0.946, NS		0.438, NS		0.388, NS	

* Wilcoxon signed-rank test.

**Table 6:** Comparison of caries occurrence in teeth sealed with conventional (Delton) and nanofilled (Filtek) resin sealant at 6 months, 12 months and 18 months evaluation.

	6 Months		12 Months		18 Months	
	Conventional	Nanofilled	Conventional	Nanofilled	Conventional	Nanofilled
Caries-free teeth	55 (94.8%)	55 (94.8%)	52 (89.7%)	53 (91.4%)	50 (86.2%)	52 (89.7%)
Caries development ^a^	3 (5.2%)	3 (5.2%)	6 (10.3%)	5 (8.6%)	8 (13.8%)	6 (10.3%)
*p* value *	0.9, NS		0.9, NS		0.727, NS	

^a^ includes filled teeth. * McNemar test.

## Data Availability

Data available on request to maintain confidentiality. The data presented in this study are available on request from the PI (First author). The data are not publicly available due to detailed information of the participants present in the data.
